# Mesoscopic superstructures of flexible porous coordination polymers synthesized *via* coordination replication†
†Electronic supplementary information (ESI) available: Full experimental procedures, powder X-ray diffraction data, thermogravimetric analysis data, adsorption isotherms, scanning electron microscopy data, and additional discussion. See DOI: 10.1039/c5sc02034d



**DOI:** 10.1039/c5sc02034d

**Published:** 2015-06-29

**Authors:** Kenji Sumida, Nirmalya Moitra, Julien Reboul, Shotaro Fukumoto, Kazuki Nakanishi, Kazuyoshi Kanamori, Shuhei Furukawa, Susumu Kitagawa

**Affiliations:** a Institute for Integrated Cell-Material Sciences (WPI-iCeMS) , Kyoto University , Yoshida, Sakyo-ku , Kyoto 606-8501 , Japan . Email: shuhei.furukawa@icems.kyoto-u.ac.jp ; Email: kitagawa@icems.kyoto-u.ac.jp; b Department of Chemistry , Graduate School of Science , Kyoto University , Kitashirakawa, Sakyo-ku , Kyoto , 606-8502 , Japan

## Abstract

The coordination replication technique is employed for the direct conversion of a macro- and mesoporous Cu(OH)_2_–polyacrylamide composite to three-dimensional superstructures consisting of the flexible porous coordination polymers, Cu_2_(bdc)_2_(MeOH)_2_ and Cu_2_(bdc)_2_(bpy) (bdc^2–^ = 1,4-benzenedicarboxylate, bpy = 4,4′-bipyridine). Detailed characterization of the replicated systems reveals that the structuralization plays an important role in determining the adsorptive properties of the replicated systems, and that the immobilization of the crystals within a higher-order architecture also affects its structural and dynamic properties. The polyacrylamide polymer is also found to be crucial for maintaining the structuralization of the monolithic systems, and in providing the mechanical robustness required for manual handling. In all, the results discussed here demonstrate a significant expansion in the scope of the coordination replication strategy, and further confirms its utility as a highly versatile platform for the preparation of functional three-dimensional superstructures of porous coordination polymers.

## Abstract 2

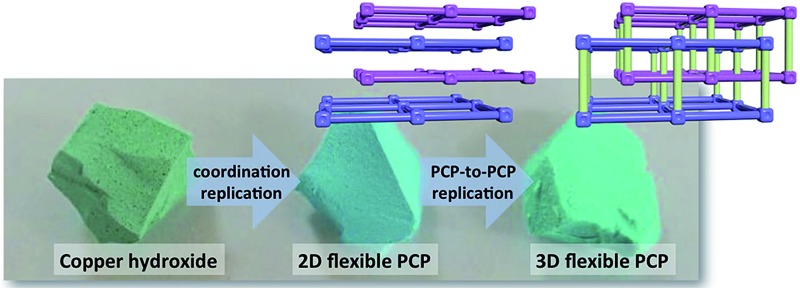
Monolithic superstructures of two- and three-dimensional flexible frameworks are prepared *via* coordination replication from a copper hydroxide parent phase.

## Introduction

The design and synthesis of porous coordination polymers (PCPs) or metal–organic frameworks (MOFs) has experienced an intensive focus in recent years,^1^ due to their potential use in applications such as gas storage, molecular separations, and heterogeneous catalysis.^2^ These compounds are assembled from metal-containing nodes bridged by organic linkers, which form porous structures that are characterized by high surface areas, as well as tunable pore dimensions and pore surface chemistry. While the ability to conveniently construct new materials from the combination of a metal salt and organic ligand (in the so-called modular approach) has provided researchers with a tremendously large library of compounds, there is an urgent need for versatile synthetic strategies for the convenient fabrication of PCPs in a structuralized form.^3^ Here, synthetic routes have begun to emerge for the bottom-up preparation of zero- (*e.g.* hollow spheres), one- (rods), two- (films), and three-dimensional (monolithic) superstructures of PCPs.^3a,b^ A feature common to the preparative methodologies of the systems reported so far is that they provide a precise control of the crystallization interface at which PCP formation occurs, resulting in the precipitation of the PCP with the desired structuralized architecture.

An elegant technique that has recently emerged for the preparation of three-dimensional superstructures of PCPs is the so-called *coordination replication* strategy.^4–7^ In this method, a structuralized metal source (such as a metal oxide) is employed as a template, which undergoes conversion in a ligand solution into a three-dimensional PCP superstructure with retention of the original structure. While this technique has been successfully demonstrated with a small number of PCP systems so far,^4^ investigations of the incorporation of molecular-scale flexibility within structuralized systems with sophisticated dynamic properties are yet to emerge. While studies of this type are of high interest from a fundamental perspective due to the prospects of new phenomena emerging from the embedding of such dynamic building blocks in a structuralized form, the identification of suitable starting materials and PCP systems is challenging due to the difficulty in preparing metal-based compounds in well-defined structures, as well as the currently limited scope of structuring techniques.

**Fig. 1:**
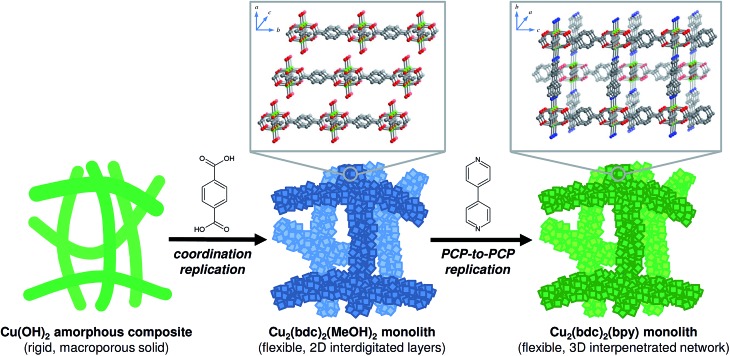
A conceptual illustration summarizing the two-step replication procedure employed in this work. In the first step, a macro- and mesoporous Cu(OH)_2_–polyacrylamide (PAAm) composite is subjected to a coordination replication process *via* treatment with H_2_bdc (bdc^2–^ = 1,4-benzenedicarboxylate), resulting in a monolith consisting of the two-dimensional layered framework, Cu_2_(bdc)_2_(MeOH)_2_. During this step, there is a significant increase in the internal solid volume (*versus* void volume) due to the Cu_2_(bdc)_2_(MeOH)_2_ crystals occupying a much greater volume compared to the precursor. In the actual monolith, this largely eliminates the macroporosity within the structure while keeping the external macroscopic dimensions. In the second step, the obtained monolith is subjected to a PCP-to-PCP replication procedure in the presence of 4,4′-bipyridine (bpy), which leads to the pillaring of the two-dimensional layers and formation of a monolith constructed from the three-dimensional, interpenetrated Cu_2_(bdc)_2_(bpy) framework. Inset: portions of the structures of each of the PCP compounds (one half of the interpenetrated framework of Cu_2_(bdc)_2_(bpy) is shown faded). Green, grey, blue, and red spheres represent Cu, C, N, and O atoms, respectively. H atoms, and solvent molecules (except for the directly coordinated atom) have been omitted for clarity.

In this work, we address these challenges *via* the structuring of flexible copper-based PCPs, namely Cu_2_(bdc)_2_(MeOH)_2_, which has a two-dimensional interdigitated structure, and Cu_2_(bdc)_2_(bpy), which comprises a three-dimensional interpenetrated structure, into three-dimensional monolithic superstructures (bdc^2–^ = 1,4-benzenedicarboxylate, bpy = 4,4′-bipyridine).^8^ A macro- and mesoporous Cu(OH)_2_–polyacrylamide (PAAm) monolithic material was chosen as a precursor for the coordination replication strategy, which was firstly successfully converted into a Cu_2_(bdc)_2_(MeOH)_2_ monolith (“daughter” phase), followed by a PCP-to-PCP replication to fabricate a Cu_2_(bdc)_2_(bpy) monolith (“granddaughter” phase) *via* the pillar ligand (bpy) insertion process (see Fig. 1). Importantly, unique adsorptive and dynamic properties are observed following immobilization of the PCPs within the three-dimensional superstructures, and the potential origins of these effects are discussed in the context of both the composition and the structures of the monoliths.

## Experimental section

### General considerations

Unless otherwise noted, all reagents were obtained from commercial vendors and used as received. While all syntheses were carried out in the air, the desolvated forms of each of the compounds were handled and stored in a nitrogen-filled glove box. Solvothermal syntheses were carried out in a DKN302 constant temperature oven (Yamato Scientific Co., Ltd) using glass vials sealed with Teflon-lined lids. Nitrogen and methanol adsorption measurements were carried out on a BELSORP-max adsorption analyser (BEL Japan, Inc.) equipped with a constant temperature bath. Powder X-ray diffraction patterns were collected using a Smartlab X-ray Diffractometer (Rigaku Corp.) equipped with a Cu Kα source.

### Field-emission scanning electron microscopy (FE-SEM)

Scanning electron microscopy (SEM) images were collected using a JEOL JSM-7001F4 electron microscope. Powder and monolith samples were evacuated to remove any residual solvent molecules, and attached to a 13.5 mm substrate using double-sided carbon tape. The samples were then coated with osmium nanoparticles to a thickness of 5 nm, and transferred to the SEM instrument. The images were collected using an emission voltage between 10 and 15 kV.

### Synthetic procedures

#### Cu(OH)_2_–polyacrylamide monolith

The parent phase was synthesized by sol–gel processing as reported recently,^9^ using a starting mixture of CuCl_2_·2H_2_O (1.53 g, 8.97 mmol), polyacrylamide (PAAm; 0.60 g, *M*_w_ ∼ 10 000), water (1.10 mL), ethanol (0.30 mL), glycerol (2.40 mL), and propylene oxide (1.47 mL, 21.0 mmol). The as-synthesized form of the monolith was stored in 2-propanol, and was rinsed with methanol prior to the coordination replication procedure. Note that after washing, some Cl^–^ ions still remain in the composition (*ca.* 4 wt%),^9^ but we refer to the starting structure as “Cu(OH)_2_–polyacrylamide monolith” for simplicity.

#### Bulk Cu_2_(bdc)_2_(bpy)

To a 500 mL round-bottom flask, H_2_bdc (210 mg, 1.26 mmol) and methanol (200 mL) were added, and the mixture was refluxed under Ar for 2 h. After this time, a commercially-obtained Cu(OH)_2_ powder (121 mg, 1.24 mmol) was added, and the solution was refluxed for a further 3 days. After this time, a sky-blue precipitate was formed, and the reaction solution was cooled to room temperature. Then, a mixture of bpy (100 mg, 0.64 mmol) in methanol (100 mL) was added to the flask, and the solution was stirred vigorously for 3 days at room temperature. This induced a color change of the solid to pale-green. The resulting solid was isolated by vacuum filtration, washed with methanol (3 × 50 mL), and dried under a reduced pressure.

#### Cu_2_(bdc)_2_(MeOH)_2_–polyacrylamide monolith

**Fig. 2:**
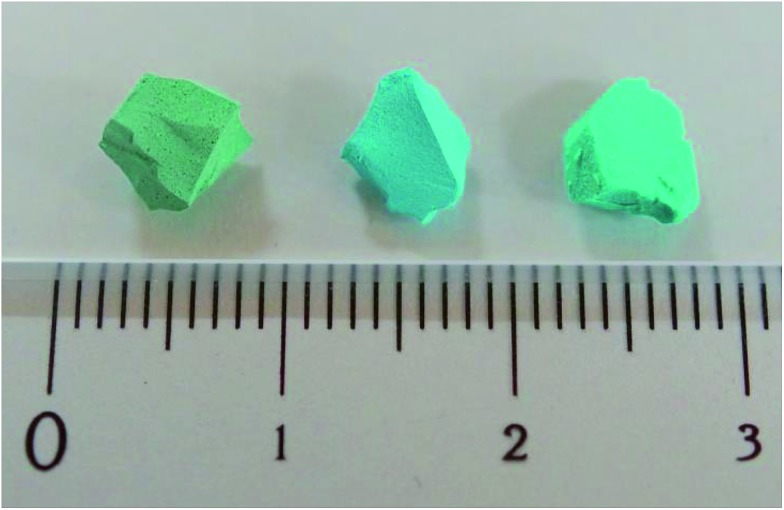
Optical images showing representative samples of (left) the Cu(OH)_2_–polyacrylamide (PAAm) composite monolith, (center) after coordination replication to form the Cu_2_(bdc)_2_(MeOH)_2_ monolith, and (right) after PCP-to-PCP replication to form the Cu(bdc)_2_(bpy) monolith.

An approximately 5 mm × 5 mm × 5 mm piece of the as-prepared Cu(OH)_2_–polyacrylamide monolith (Fig. 2, left) was inserted into a tapered glass tube (i.d. 8 mm), and placed into a 20 mL glass vial containing H_2_bdc (50 mg, 0.30 mmol) and methanol (20 mL). The vial was sealed and placed in an oven set to a temperature of 60 °C for 12 h, after which time the color of the monolith changed from green to sky-blue (see Fig. 2, center). The glass tube (containing the monolith) was then removed and placed in a fresh ligand solution of the same composition, and placed back in the oven for a further 12 h. This procedure was repeated until the total reaction time was 7 days, after which time the fully replicated monolith was washed by immersion in neat methanol (50 mL) for 24 h to remove any unreacted H_2_bdc. The washing procedure was repeated three times, and the material was stored in neat methanol to avoid degradation of the resultant structure.

#### Cu_2_(bdc)_2_(bpy)–polyacrylamide monolith

In a 100 mL glass vial, the Cu_2_(bdc)_2_(MeOH)_2_–polyacrylamide monolith obtained in the previous step was immersed in methanol (45 mL). Then, a solution of bpy (10.0 mg, 64.0 μmol) dissolved in methanol (5 mL) was slowly added, and the contents of the vial were allowed to stand undisturbed at room temperature for 24 h. Then, 5 mL of the solution was removed, replaced with a bpy solution with the same composition added initially, and the mixture was allowed to stand for a further 24 h. This procedure was repeated until the total reaction time was 5 days, after which time the color of the monolith had changed to blue-green (Fig. 2, right). The solid was washed and stored using the same method as described for the Cu_2_(bdc)_2_(MeOH)_2_–polyacrylamide monolith.

## Results and discussion

### Synthesis and characterization

The coordination replication technique is an attractive method for the structuralization of PCP materials, since a potentially wide variety of metal-based precursors can be shaped into a desired form *via* conventional fabrication techniques, such as sol–gel processing. Here, the main requirement for precursor materials is a slow dissolution rate relative to the crystallization rate of the target PCP crystals, such that crystal growth is spatially constrained at the interface between the solid precursor and the ligand solution.^4^ This represents one of the main challenges in expanding the scope of coordination replication synthesis, and precursors that offer the correct balance between solubility and reactivity under the reaction conditions for the PCP formation process have remained limited so far.

Among the solid sources containing Cu^2+^ considered for the formation of the Cu_2_(bdc)_2_(MeOH)_2_ and Cu_2_(bdc)_2_(bpy) frameworks, Cu(OH)_2_ was chosen for further study owing to its low solubility in polar organic solvents and high reactivity toward acids.^10^ Consequently, synthetic conditions for the synthesis of the two compounds were developed using a commercially-available bulk crystalline powder of Cu(OH)_2_. The screening of various parameters, including the reaction solvent, the metal-to-ligand ratio, and the reaction time revealed that the addition of a stoichiometric quantity of Cu(OH)_2_ to a refluxing solution of H_2_bdc in methanol afforded Cu_2_(bdc)_2_(MeOH)_2_ after a reaction time of 3 days. Next, a suspension of Cu_2_(bdc)_2_(MeOH)_2_ was treated with an excess of bpy in methanol, resulting in the installation of bpy pillars between every second square grid layer to produce the interpenetrated Cu_2_(bdc)_2_(bpy) framework (see Fig. S3†). SEM observation confirmed a plate-like crystal morphology (Fig. S4†), and N_2_ adsorption measurements (Fig. S5†) at 77 K gave a BET surface area^11^ of 1030 m^2^ g^–1^ (Langmuir surface area: 1300 m^2^ g^–1^) which is somewhat higher than the corresponding value of 700 m^2^ g^–1^ measured previously for a sample prepared from a conventional method that uses CuSO_4_ as the Cu^2+^ source.^8*a*^

**Fig. 3:**
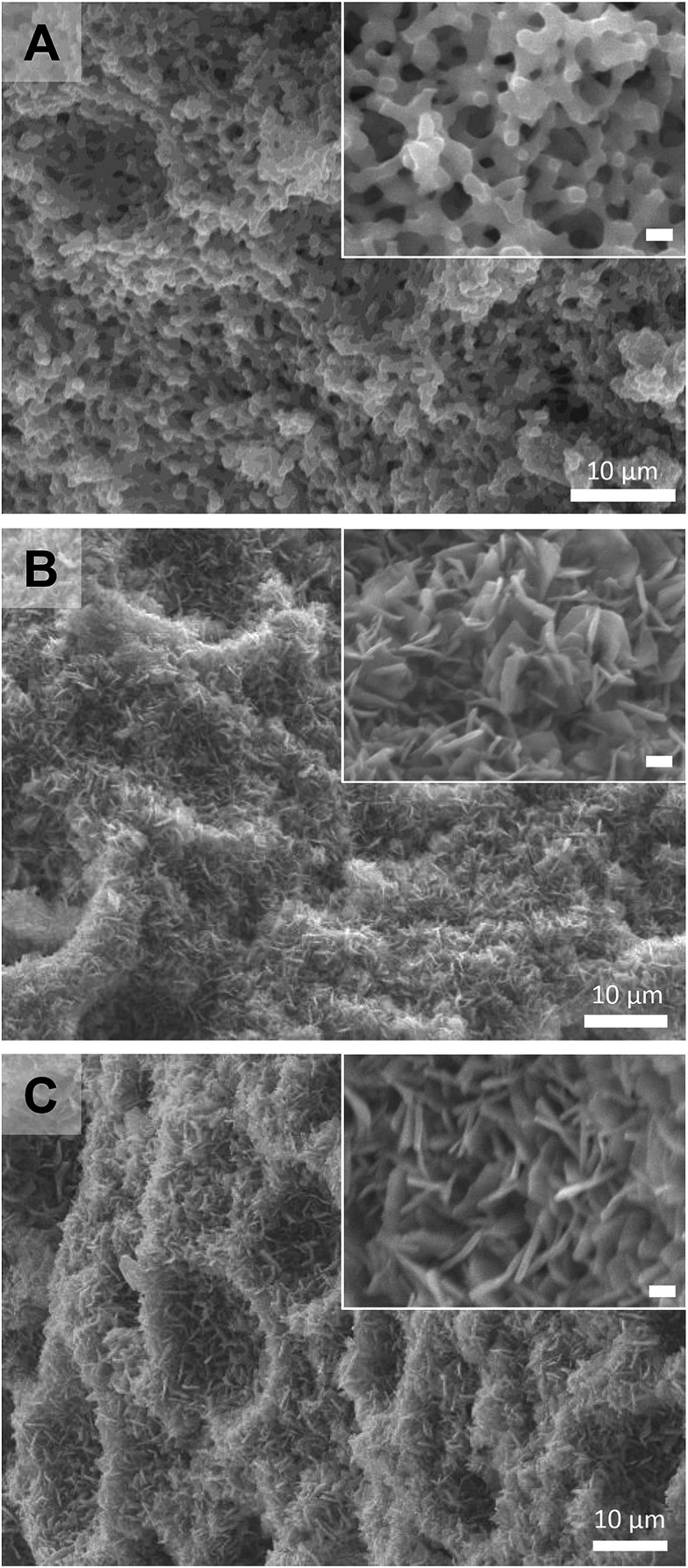
Field-emission SEM images of (A) the Cu(OH)_2_–polyacrylamide (PAAm) composite material, (B) after coordination replication to form the Cu_2_(bdc)_2_(MeOH)_2_ monolith, and (C) after PCP-to-PCP replication to form the Cu_2_(bdc)_2_(bpy) monolith. Scale bars for the inset images represent a distance of 1 μm.

Following the successful demonstration of the synthesis of Cu_2_(bdc)_2_(MeOH)_2_ and Cu_2_(bdc)_2_(bpy) from crystalline Cu(OH)_2_ powders, a structuralized form of Cu(OH)_2_ was required for coordination replication studies. Recently, a method for the preparation of an amorphous macro- and mesoporous Cu(OH)_2_–polyacrylamide composite material *via* sol–gel processing accompanied by phase separation, and its conversion to the prototypical and rigid PCP, Cu_3_(btc)_2_, was reported.^9,12^ The amorphous nature of the Cu(OH)_2_ within this parent phase is expected to have a similar (or enhanced) reactivity compared to crystalline Cu(OH)_2_, and was identified as a suitable candidate for further study. In this case, the synthetic procedure of the Cu(OH)_2_–polyacrylamide parent phase was adapted to prepare a monolithic solid featuring continuous macropores with a diameter of *ca.* 1 μm (see Fig. 2, left, and Fig. 3A). Analysis of the porosity of the parent monolith used in this work *via* N_2_ adsorption isotherms afforded a type-IV profile typical of a mesoporous solid (Fig. S6†). The determination of a pore size distribution based on this data revealed a maximum density at *ca.* 5 nm for the mesopores. Note that the large pores present within this monolith are expected to facilitate the diffusion of the organic linkers throughout the solid, which is required for full conversion during the coordination replication procedure.

The Cu(OH)_2_–polyacrylamide monolith was suspended and heated within a solution of H_2_bdc in methanol for an extended period of 7 days (with daily exchange of the mother liquor), which resulted in a color change of the solid from green to sky-blue. Importantly, the external dimensions of the monolith and its mechanical integrity were retained despite the long period of treatment (see Fig. 2, center).^13^ Observation of the surface of the monolith following replication by SEM revealed the growth of square plate-like crystals approximately 1 μm in width from the walls of the co-continuous structure (see Fig. 3B). SEM observation following slicing of a monolith sample to expose the cross-section (depth direction) of the structure showed crystals of the same morphology had uniformly formed throughout the material (see Fig. S7†), but the macropores were almost completely eliminated. This is because the conversion from Cu(OH)_2_ to Cu_2_(bdc)_2_(MeOH)_2_ results in a volume increase of approximately 10 times (based on Cu^2+^ density in the bulk, crystalline forms of both compounds). The complete conversion of the Cu(OH)_2_ of the parent phase was further confirmed by thermogravimetric analysis (TGA), which did not exhibit a weight loss at the decomposition temperature of Cu(OH)_2_ of *ca.* 80 °C (see Fig. S8†). The TGA data could also be used to estimate a polyacrylamide content of 15.0 wt%, which is close to the composition employed during the preparation of the Cu(OH)_2_–polyacrylamide monolith of *ca.* 20.0 wt%. Note that, in the preparation of the Cu_2_(bdc)_2_(MeOH)_2_ monolith, the reaction conditions developed for the preparation of bulk powders of the same compound from crystalline Cu(OH)_2_ was successfully used for monolith conversion. This agrees with our experience using the coordination replication method for the synthesis of Al-based PCP architectures from Al_2_O_3_ phases,^4^ which has demonstrated that amorphous or less-dense variants of an inorganic compound tend to dissolve faster or have higher reactivities since they have lower lattice energies. This results in the right balance between precursor dissolution and PCP crystallization, which is required for preservation of the structuring of the parent phase.

Next, the Cu_2_(bdc)_2_(MeOH)_2_ monolith was immersed in a methanol solution of bpy to induce pillaring of the square grid layers of the two-dimensional framework to afford the three dimensional Cu_2_(bdc)_2_(bpy) compound. After several hours, the color of the monolith changed from sky-blue to blue-green (see Fig. 2, right). SEM data revealed the retention of the structuralization of the monolith following replication accompanied with a slight increase in the thickness of the crystals, which is consistent with the insertion of the bpy pillars between the dinuclear copper paddlewheels of every second square grid layer (Fig. 3C). Estimation of the composition of the monolith *via* TGA data revealed a polyacrylamide content of 2.0 wt% (Fig. S16†), the loss of which, as discussed in the following section, has important consequences with respect to the properties of the monoliths.

### Structural features and structural flexibility of the replicated frameworks

#### Cu_2_(bdc)_2_(MeOH)_2_ monolith

**Fig. 4:**
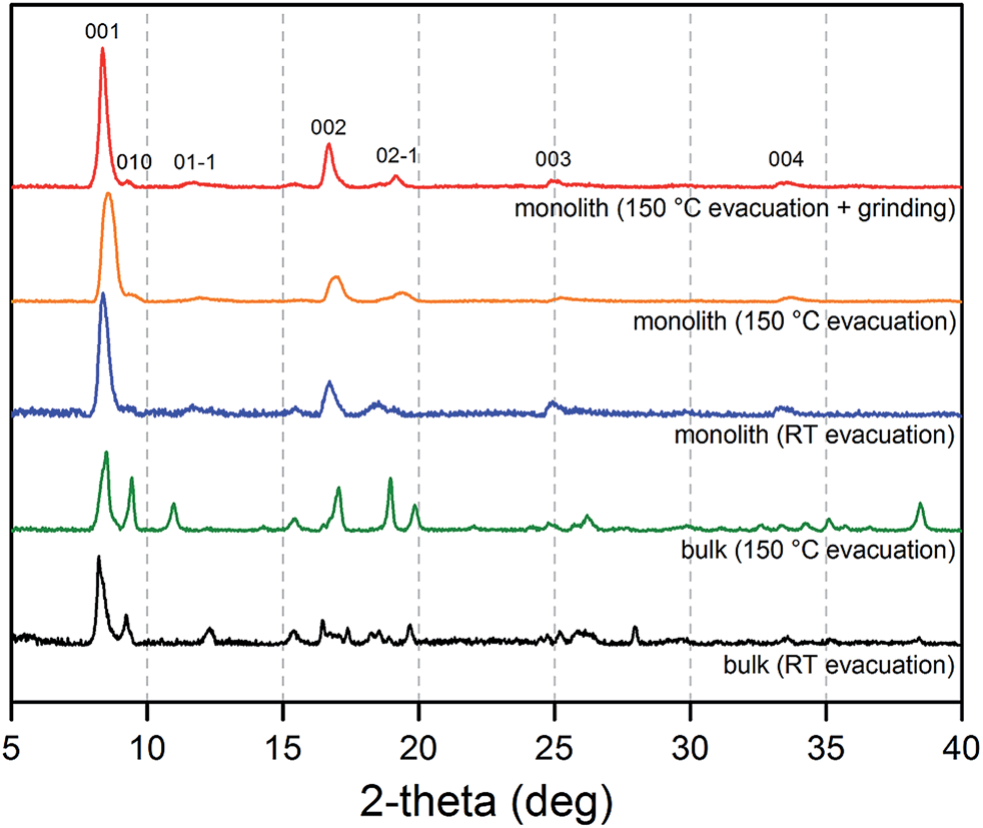
Powder X-ray diffraction patterns collected for a bulk Cu_2_(bdc)_2_(MeOH)_2_ powder after evacuation at room temperature (black) and 150 °C (green), and a Cu_2_(bdc)_2_(MeOH)_2_ monolithic sample after evacuation at room temperature (blue), 150 °C (orange), and 150 °C followed by mechanical grinding to remove the structuralization of the material (red).

The properties of the replicated monolith were probed using a combination of powder X-ray diffraction, SEM, TGA, infrared spectroscopy, and sorption experiments. Diffraction patterns obtained from a solvated fragment of the replicated solid were indicative of a highly crystalline framework phase, with reflections that were well-matched with those of solvated bulk Cu_2_(bdc)_2_(MeOH)_2_ (see Fig. 4). Surprisingly, a significant number of peaks were absent in the diffraction pattern of the monolithic phase. Assignment of the diffraction peaks observed for the monolith revealed that the 0*k*0, 00*l*, and 0*kl* reflections were present, while all reflections with a non-zero *h* component were significantly broadened or absent.^14^ The structure of the Cu_2_(bdc)_2_(MeOH)_2_ compound is such that the crystallographic *a*-axis (*i.e.* the *h*00 reflection) represents the periodicity of the stacking of the two-dimensional square grids (see Fig. 1), and the absence of these reflections can be attributed to its disruption (or “amorphization”) upon integration into the monolith. This is analogous to a phenomenon observed in carbon-based materials with a turbostratic structure, in which 00*l* reflections are prominently observed (with a broadened peak width) compared to its crystalline counterpart, graphite.^15^

**Fig. 5:**
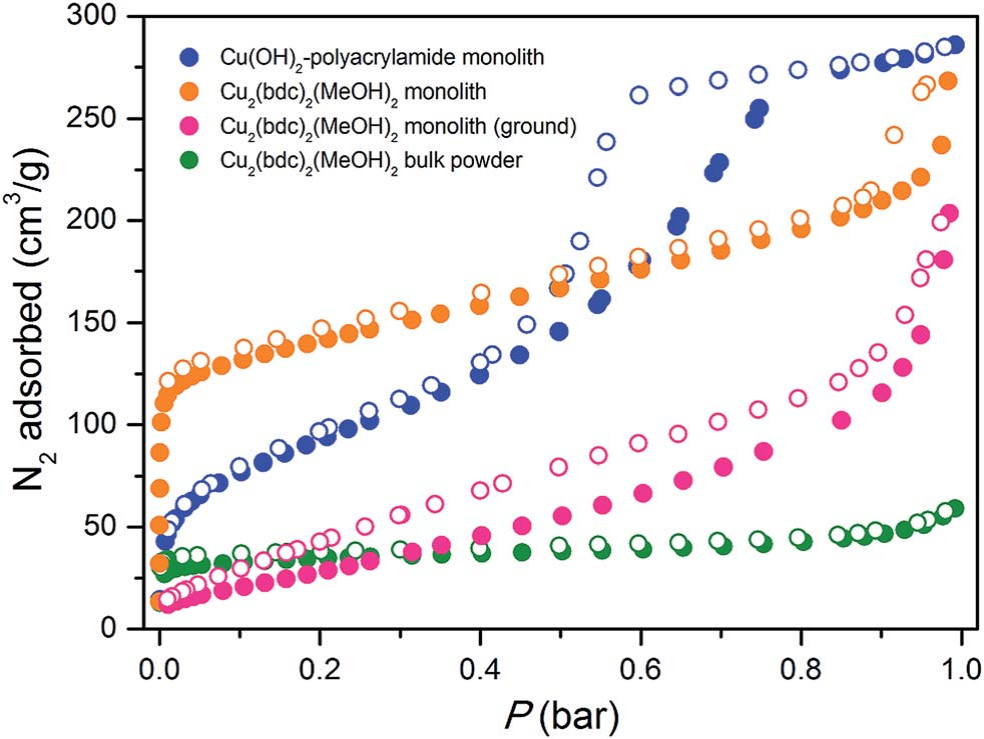
Nitrogen adsorption isotherms collected at 77 K for the parent Cu(OH)_2_ monolith prior to replication (blue), a bulk Cu_2_(bdc)_2_(MeOH)_2_ powder (green), a Cu_2_(bdc)_2_(MeOH)_2_ monolith prepared by coordination replication (orange), and the Cu_2_(bdc)_2_(MeOH)_2_ monolith after grinding into a uniform powder to eliminate the effect of structuralization (pink). Closed and open symbols represent adsorption and desorption data, respectively.

The origins of this unusual feature of the powder X-ray diffraction data were further probed by N_2_ adsorption analysis at 77 K after activation of the monolith at 150 °C.^16^ Fig. 5 displays data collected for the parent Cu(OH)_2_–polyacrylamide monolith, the Cu_2_(bdc)_2_(MeOH)_2_ monolith and a bulk Cu_2_(bdc)_2_(MeOH)_2_ powder sample. Remarkably, while the bulk material showed a negligible N_2_ uptake owing to the inability of N_2_ to open and access the interlayer spacing, the structuralized variant exhibited significant uptake at low pressures, reminiscent of a type-I isotherm observed for a microporous solid. Indeed, a BET analysis of the sorption data (see Fig. S9†) afforded a surface area of 520 m^2^ g^–1^,^17^ which is significantly greater than can be accounted for by the sorption properties of the parent Cu(OH)_2_–polyacrylamide phase and bulk Cu_2_(bdc)_2_(MeOH)_2_. This suggests that the structural influence of the interactions between the Cu_2_(bdc)_2_(MeOH)_2_ crystals and the polyacrylamide chains at the molecular scale in turn impart considerably different sorption properties to the PCP phase compared to its bulk counterpart.

The powder diffraction and adsorption data observed here can be reconciled by considering the role of the polyacrylamide polymer in the replicated system. The polyacrylamide content of the Cu_2_(bdc)_2_(MeOH)_2_ monolith of approximately 15.0 wt% is a component required for the integrity of the three-dimensional structuralization. Here, it is expected that the anchoring of the crystals to the polymer occurs by way of Cu^2+^–amide interactions, which inherently requires the polymer to become partially incorporated between the layers of the framework (*i.e.* by coordination to the dinuclear paddlewheels). This is expected to disrupt the periodicity of the PCP in the crystallographic *a*-direction of the framework (while leaving the crystallinity of the *bc* plane unaffected), and the creation of uneven spacings between the square grid layers, some of which are sufficiently large for N_2_ to be incorporated at low temperatures. This phenomenon is unique to Cu_2_(bdc)_2_(MeOH)_2_ in a structuralized state, since such points of anchoring do not exist in the bulk form. Further, it demonstrates the importance of molecular scale interactions between the PCP crystals and the support in determining the adsorptive and dynamic behavior of the system as a whole.

The impact of structuralization in the Cu_2_(bdc)_2_(MeOH)_2_ system was further investigated by destroying the architecture by mechanical grinding of the monolith into a fine powder. Although the crystallinity of the sample was preserved following this process (see Fig. 4), N_2_ adsorption data at 77 K revealed the complete loss of microporosity once in a ground powder form (see Fig. S10†). This can be ascribed to the pulverization of the crystals as observed by SEM (Fig. S11†), which leads to most of the crystalline fragments no longer being bound by the polyacrylamide polymer. Indeed, while the microporous region of the N_2_ isotherm no longer shows a significant uptake, the profile exhibits a monotonic increase up to 190 cm^3^ g^–1^ at a pressure of 1 bar, consistent with surface adsorption of N_2_ to the polyacrylamide polymer surface. In addition, preparation of Cu(bdc)_2_(MeOH)_2_ from a uniformly ground sample of the Cu(OH)_2_–polyacrylamide parent phase (prepared under the same reaction conditions as bulk Cu(bdc)_2_(MeOH)_2_) yielded a sample of the same composition as the Cu(bdc)_2_(MeOH)_2_ monolith. However, unlike the monolith form, the material displays little microporosity despite the presence of polyacrylamide in the overall composition (see Fig. S12 and S13†). This further supports the observation that the immobilization of the Cu(bdc)_2_(MeOH)_2_ crystals within the three-dimensional architecture provides the additional microporosity observed here.

#### Cu_2_(bdc)_2_(bpy) monolith

**Fig. 6:**
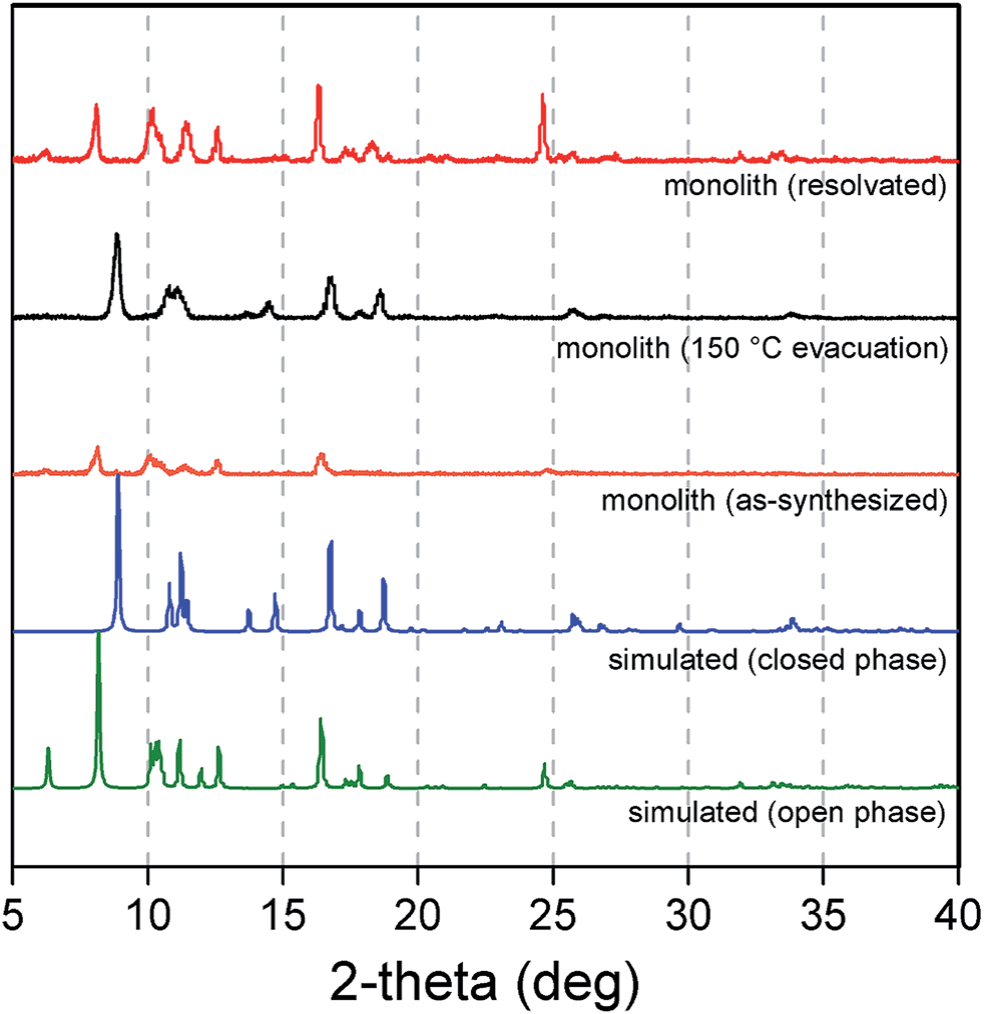
Powder X-ray diffraction patterns simulated for the open (green) and closed (blue) forms of Cu_2_(bdc)_2_(bpy), and experimental patterns for an as-synthesized sample of a Cu_2_(bdc)_2_(bpy) monolith (orange), after evacuation at 150 °C (black), and resolvation by immersion in methanol (red).

**Fig. 7:**
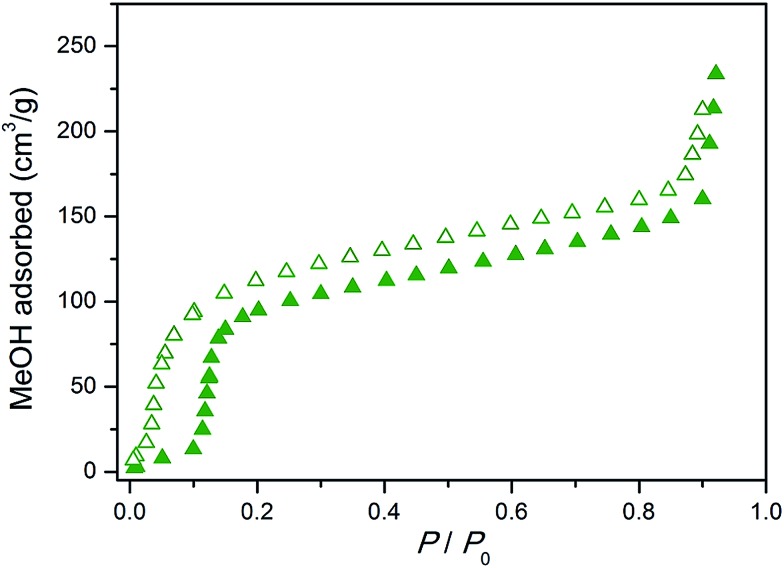
Methanol adsorption isotherm collected at 298 K for the Cu_2_(bdc)_2_(bpy) monolith. Closed and open symbols represent adsorption and desorption, respectively.

The composition, structure, and framework flexibility of the replicated monolith was characterized using a combination of powder X-ray diffraction, SEM, and adsorption experiments. As shown in Fig. 6, powder X-ray diffraction data collected for an as-synthesized sample afforded reflections corresponding to the open pore form of the framework simulated from single-crystal data. *In situ* activation of the sample under a He flow at 150 °C led to a structural change in the framework to the corresponding closed pore form, which is consistent with the removal of the methanol molecules within the pores. Resolvation of the material in methanol resulted in a return to the open form phase with retention of the three-dimensional superstructure. Note that this solvation–desolvation process could be repeated several times without loss of the integrity of the monolith, demonstrating the successful preparation of a monolithic structure consisting of reversibly flexible building blocks. A methanol isotherm collected for an activated sample (see Fig. 7) exhibited a stepped isotherm with hysteresis in the desorption branch, which is typical for a gate-opening type structural transition of the framework. Comparison of the methanol uptake over several cycles showed no degradation to the adsorption profile (Fig. S14†), confirming the stability of the monolith with respect to flexing of the framework.

Next, the effect of structuralization of the Cu_2_(bdc)_2_(bpy) compound in a monolith form was probed by comparing its methanol adsorption isotherm after mechanical grinding of the framework. Surprisingly, in contrast to the Cu_2_(bdc)_2_(MeOH)_2_ monolith, little change was observed after the destruction of the structuralization with regard to both the gate-opening pressure and the quantity of methanol adsorbed (Fig. S15†). Furthermore, comparison with a bulk powder of Cu_2_(bdc)_2_(bpy) also revealed an almost identical adsorption profile, revealing that both the structural flexibility and the adsorption properties of the monolith are a good match to those of a bulk sample of the same compound. This is a somewhat surprising result given that, based on the unusual properties observed for the Cu_2_(bdc)_2_(MeOH)_2_ monolithic system, the immobilization of the Cu_2_(bdc)_2_(bpy) crystals in a monolith form might be expected to influence the adsorptive and dynamic properties of the system.

**Fig. 8:**
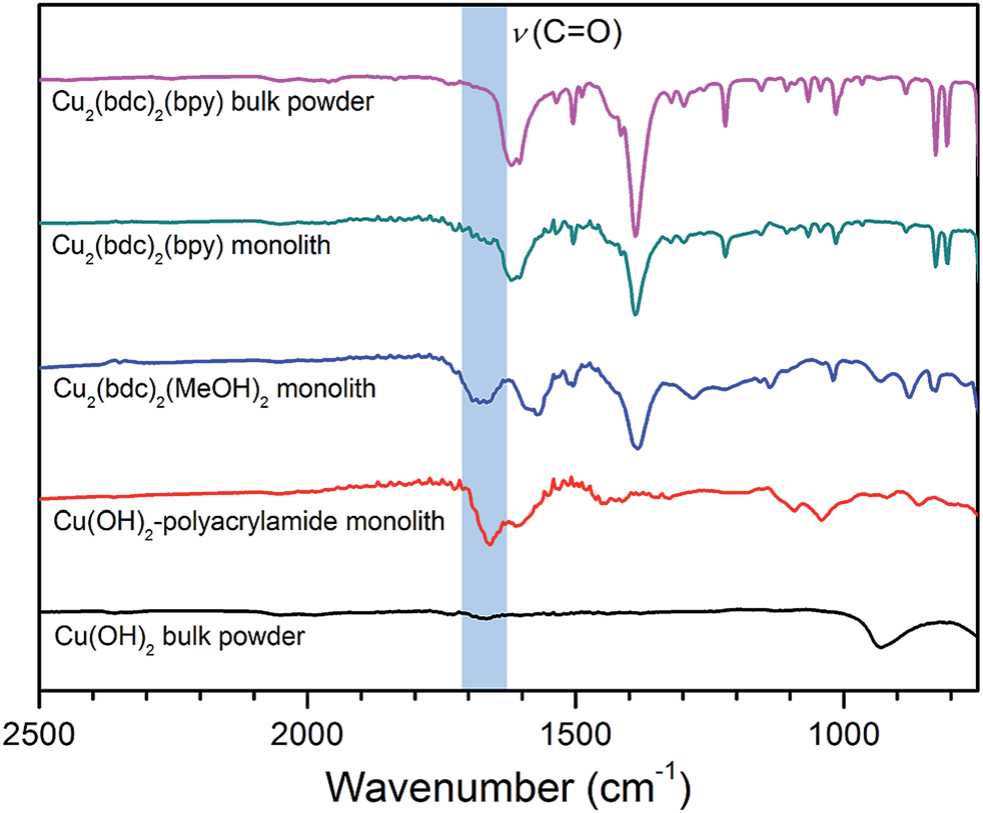
Infrared spectra for bulk Cu(OH)_2_ (black), the parent Cu(OH)_2_ monolith (red), the Cu_2_(bdc)_2_(MeOH)_2_ monolith prepared by coordination replication (blue), the Cu_2_(bdc)_2_(bpy) monolith prepared by PCP-to-PCP replication (green), and a bulk Cu_2_(bdc)_2_(bpy) powder (pink).

In order to elucidate the origin of this result, IR and TGA data were collected to evaluate the composition of the Cu_2_(bdc)_2_(bpy) replicate. As is clear from the IR data presented in Fig. 8, the spectrum observed for the activated form of the Cu_2_(bdc)_2_(bpy) monolith shows a close match with that of a bulk sample of the same framework. However, in comparison with the parent and Cu_2_(bdc)_2_(MeOH)_2_ monolith, the C–N stretch at approximately 1660 cm^–1^ originating from the amide moiety of the polyacrylamide polymer is greatly diminished, suggesting that the polymer component is excluded from the structure during the insertion of the bpy pillars. This was further confirmed by the TGA data shown in Fig. S16,† which allowed the polyacrylamide content to be calculated as 2.0 wt%, compared with 20.0 wt% and 15.0 wt% in the parent Cu(OH)_2_–polyacrylamide and Cu_2_(bdc)_2_(MeOH)_2_ monoliths, respectively. The loss of polyacrylamide from the structure is also consistent with a decrease in the mechanical robustness of the Cu_2_(bdc)_2_(bpy) monolith, emphasizing its key role in providing the effect of structuralization of the monolithic structure following replication.

The origin of the loss of polyacrylamide from the structure was probed *via* a number of control experiments. Immersion of the parent Cu(OH)_2_–polyacrylamide and Cu_2_(bdc)_2_(MeOH)_2_ monoliths in methanol resulted in no change to the composition or the structuralization, which provided clear evidence of the stability of the monoliths (and its associated polymer content) under these conditions. Furthermore, immersion of the parent Cu(OH)_2_–polyacrylamide compound in a methanol solution of bpy resulted in no loss in the polyacrylamide component from the structure as evaluated by TGA data (Fig. S20†). Thus, the polyacrylamide is only lost when the Cu_2_(bdc)_2_(MeOH)_2_ undergoes pillaring by the bpy molecules during the second PCP-to-PCP replication step. While an exact mechanism for the loss of polyacrylamide is not yet available, a plausible sequence of events is as follows. In the conversion of the Cu(OH)_2_–polyacrylamide monolith to the Cu_2_(bdc)_2_(MeOH)_2_ replicate, the polyacrylamide directly binds to the Cu_2_(bdc)_2_(MeOH)_2_ framework *via* amide sidechains as described above. This leads to the polymer chains, which are originally buried beneath a colloidal network of Cu(OH)_2_ particles, to become exposed after replication. This is due in part to the plate-shaped crystals of Cu_2_(bdc)_2_(MeOH)_2_ that are not expected to uniformly protect the polymer chains from access at the molecular scale. Then, upon exposure of the monolith to a solution containing bpy, the amide moieties are displaced from the Cu^2+^ centers, leaving the chains unbound and susceptible to dissolution out of the monolith. This dissolution process may additionally be assisted by a partial hydrolysis of the polymer chains, which is known to occur in the presence of basic species. Note that analysis of the reactant solution by IR and ^1^H NMR did not reveal the presence of free acrylamide monomers, suggesting a complex decomposition pathway for the PAAm component into a variety of products. As such, after the removal of the polyacrylamide component from the monolith, the limited intergrowth between the Cu_2_(bdc)_2_(bpy) crystals leads to the structural and sorption properties of the monolith largely reflecting those of a bulk powder, despite the retention of the monolithic structure.

## Conclusions and future outlook

The foregoing results have detailed the synthesis and properties of three-dimensional superstructures consisting of the flexible Cu_2_(bdc)_2_(MeOH)_2_ and Cu_2_(bdc)_2_(bpy) frameworks *via* coordination replication from a structuralized macro- and mesoporous Cu(OH)_2_–polyacrylamide composite parent phase. The synthesis of these monolithic systems expands on the scope of the coordination replication technique to include flexible PCPs, but perhaps more importantly, provides monolithic systems that exhibit properties that differ from bulk powders as a result of structuralization. In the case of the Cu_2_(bdc)_2_(MeOH)_2_ system, the anchoring of the two-dimensional framework by the polyacrylamide polymer leads to their immobilization within the superstructure, but also results in an amorphization of the interlayer direction of the framework structure. This provides the framework with an ability to adsorb N_2_, which is not observed in the absence of structuralization. For the Cu_2_(bdc)_2_(bpy) system, the framework flexibility is preserved after immobilization, leading to a flexible monolith system. In this case, the sorption and dynamic properties largely reflect the characteristics of the bulk form owing to the dissolution of the polymer phase during the PCP-to-PCP replication step. This emphasizes the importance of the polymer phase in maintaining the connectivity between crystals and in providing the system with the effects of structuralization.

The results presented here further demonstrate the versatility of the coordination replication technique, and it is envisaged that a greater library of structuralized PCPs will emerge in the near future for specific applications in areas such as molecular separations and heterogeneous catalysis. In addition, the new properties observed for the structuralized forms of the compounds suggest that new, rich phenomena could emerge as a result of detailed studies of this type. However, as revealed here, there is an urgent need for preparative routes to new parent materials that are optimized for coordination replication, and care is also needed in the selection of the target PCP system. Specifically, a high degree of crystal intergrowth is desired in order to achieve cooperative effects stemming from material structuralization. While the polyacrylamide polymer serves as an adhesive between the crystals in this case, greater intergrowth between the PCP crystals themselves would preclude the need for the use of a composite system. For example, optimization of both the crystal size (*i.e.* smaller crystals) and morphology (*i.e.* block-shaped crystals) of the PCP phase is expected to facilitate a greater preservation of the original structure of the parent material with a greater degree of intergrowth. Such optimizations of the crystal parameters have already appeared in the case of bulk crystals *via* the coordination modulation technique,^18^ and studies using this strategy for the fabrication of three-dimensionally structuralized systems composed of other functional PCP systems are already underway.

## References

[cit1] Yaghi O. M., O'Keeffe M., Ockwing N. W., Chae H. K., Eddaoudi M., Kim J. (2003). Nature.

[cit2] (b) Zhou H.-C. Kitagawa S., Chem. Soc. Rev., 2014, 43 , 5415 , , and references therein . 25011480 10.1039/c4cs90059f

[cit3] Carné-Sánchez A., Imaz I., Stylianou K. C., Maspoch D. (2014). Chem.–Eur. J..

[cit4] Reboul J., Furukawa S., Horike N., Tsotsalas M., Hirai K., Uehara H., Kondo M., Louvain N., Sakata O., Kitagawa S. (2012). Nat. Mater..

[cit5] Khaletskaya K., Reboul J., Meilikhov M., Nakahama M., Diring S., Tsujimoto M., Isoda S., Kim F., Kamei K., Fischer R. A., Kitagawa S., Furukawa S. (2013). J. Am. Chem. Soc..

[cit6] Okada K., Ricco R., Tokudome Y., Styles M. J., Hill A. J., Takahashi M., Falcaro P. (2013). Adv. Funct. Mater..

[cit7] Stassen I., Campagnol N., Fransaer J., Vereecken P., De Vos D., Ameloot R. (2013). CrystEngComm.

[cit8] Seki K. (2002). Phys. Chem. Chem. Phys..

[cit9] Fukumoto S., Nakanishi K., Kanamori K. (2015). New J. Chem..

[cit10] Nytko E. A., Helton J. S., Müller P., Nocera D. G. (2008). J. Am. Chem. Soc..

[cit11] Walton K. S., Snurr R. Q. (2007). J. Am. Chem. Soc..

[cit12] Moitra N., Fukumoto S., Reboul J., Sumida K., Zhu Y., Nakanishi K., Furukawa S., Kitagawa S., Kanamori K. (2015). Chem. Commun..

[cit13] Attempts to prepare monoliths of a sufficient size for mechanical strength measurements of the Cu_2_(bdc)_2_(MeOH)_2_ monolith were not successful in this case. While large monoliths (cylindrical tablets with a diameter of 1 cm and a height of 0.5 cm) of the Cu_3_(btc)_2_ framework were readily prepared within 30 min from the same starting precursor, 12 the conversion was found to be significantly slower in the case of Cu_2_(bdc)_2_(MeOH)_2_. The use of starting monoliths of a sufficient size resulted in samples with unreacted cores even after 14 days, likely due to preferential crystal growth at the exterior of the monolith resulting in macropore blockage, preventing diffusion of the organic linker throughout the solid. The significantly different behavior of the two systems highlights potential differences in both the molecular scale replication mechanism and the nature of the crystal growth, which are areas worthy of systematic investigation in order to optimize precursor design for specific PCP systems

[cit14] Such effects are often observed in oriented samples or those with highly anisotropic crystal shapes, although this is not expected for the replicated phase studied here due to the random distribution of spatial orientations of the crystals within the monolith

[cit15] Hishiyama Y., Nakamura M. (1995). Carbon.

[cit16] Note that this slightly lower activation temperature than for bulk powder samples allows the polyacrylamide component to be stably maintained within the framework, while allowing full removal of the methanol within the pores and bound to the Cu^2+^ ions of the dinuclear paddlewheel units

[cit17] The macroporosity is largely eliminated and the mesoporosity significantly diminished upon replication, which is due to the Cu_2_(bdc)_2_(MeOH)_2_ crystals occupying a (up to 10 times) greater volume compared to the original Cu(OH)_2_ component based on the density of Cu^2+^ ions in the respective crystal structures. The plate-like morphology of the framework crystals may also provide a less contoured surface providing fewer cavities in the mesopore length scale

[cit18] Tsuruoka T., Furukawa S., Takashima Y., Yoshida K., Isoda S., Kitagawa S. (2009). Angew. Chem., Int. Ed..

