# Supplementary material for: Mesoscopic superstructures of flexible porous coordination polymers synthesized via coordination replication
Source: Chem Sci. 2015 Jun 29;6(10):5938–46. doi: 10.1039/c5sc02034d (PMC5523079; doi:10.1039/c5sc02034d)
Supplement: Supplementary file 1 [file SC-006-C5SC02034D-s001.pdf]

Electronic Supplementary Material (ESI) for Chemical Science.
This journal is © The Royal Society of Chemistry 2015

Supporting Information for:

Mesoscopic superstructures of flexible porous coordination polymers synthesized *via* coordination replication

Kenji Sumida,[a] Nirmalya Moitra,[b] Julien Reboul,[a] Shotaro Fukumoto,[b] Kazuki Nakanishi,[b] Kazuyoshi Kanamori,[b] Shuhei Furukawa,*[a] and Susumu Kitagawa*[a]

Structure and Flexibility of the Cu(bdc)(MeOH) and Cu(bdc)(bpy)$_{0.5}$ Frameworks

The structure of the Cu(bdc)(MeOH) framework shown in Fig. S1 is one consisting of two-dimensional square grids consisting of dinuclear Cu^{2+} paddlewheels bridged by the bdc^{2-} ligands, with MeOH solvent molecules occupying the axial binding sites. The framework exhibits the reversible accommodation of guest molecules in the interlayer spaces, which results in a change in the distance between adjacent layers. The installation of bpy units results in the formation of the microporous Cu(bdc)(bpy)$_{0.5}$ compound *via* the displacement of the coordinated MeOH molecules, which features an interpenetrated structure in which the pillars span the axial Cu^{2+} sites of every second layer such that the pillars are threaded through the cavities of the square grids. The Cu(bdc)(bpy)$_{0.5}$ compound features a reversible transition between a closed phase in the evacuated state, to an open phase in the solvated state (see Fig. S2).

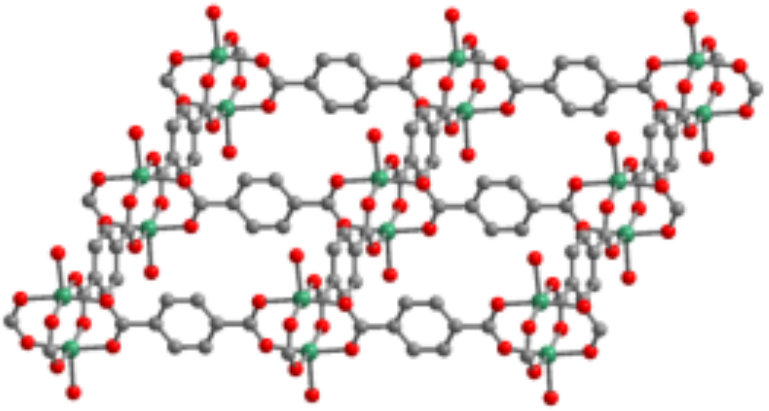

Fig. S1 A structural model showing a portion of the structure of the Cu(bdc)(MeOH) compound. Green, gray and red spheres represent Cu, C and O atoms, respectively, while H atoms and the methyl group of the coordinated methanol molecules the have been omitted for clarity.

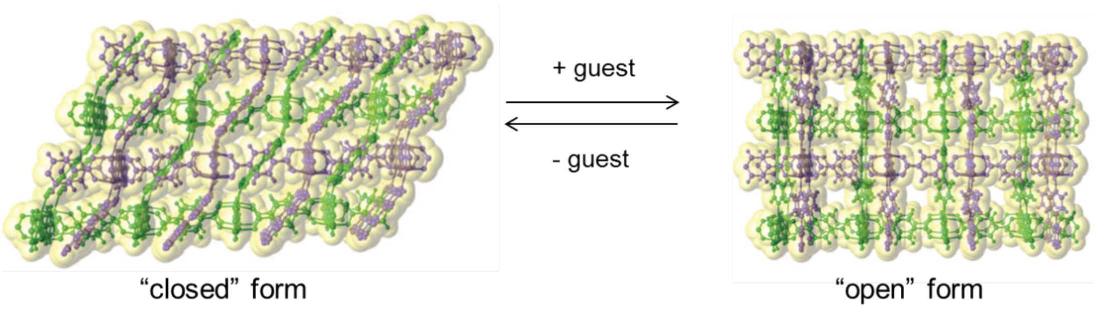

Fig. S2 Diagrams showing the interpenetrated structures of Cu(bdc)(bpy)$_{0.5}$ (purple and green), and the structural transition between closed and open pore forms upon guest inclusion and removal.

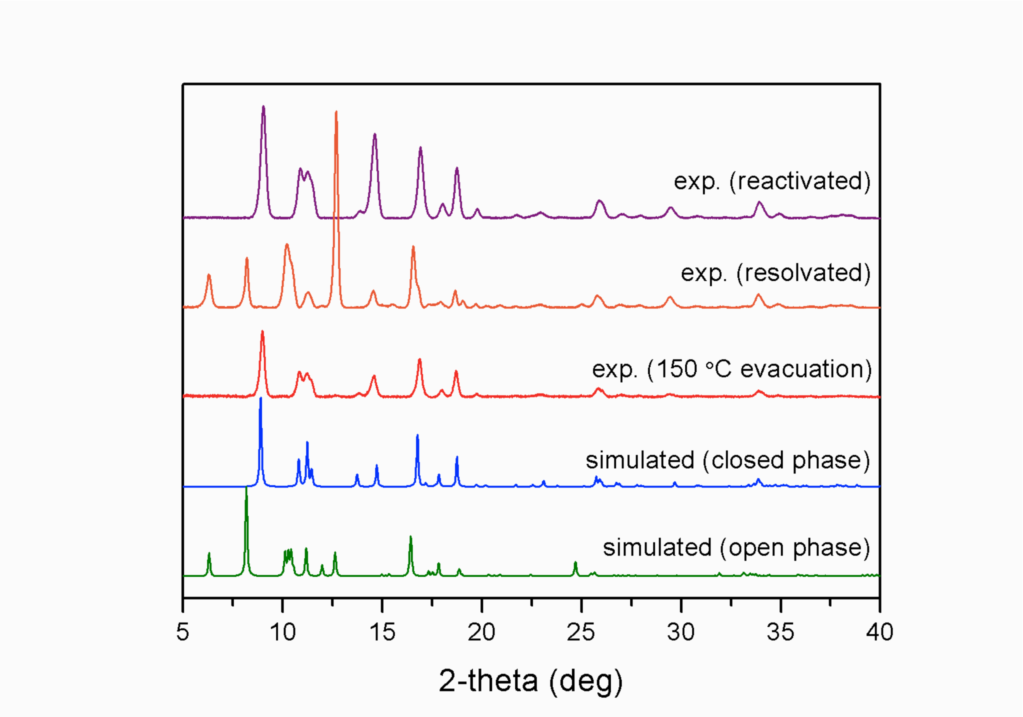

Fig. S3 Powder X-ray diffraction patterns as simulated for the open (green) and closed (blue) phases of $Cu_2(bdc)_2(bpy)$, and experimental data for a $Cu(OH)_2$-derived $Cu_2(bdc)_2(bpy)$ sample after 150 °C evacuation (red), resolvation in methanol (orange), and reactivation at 150 °C (purple).

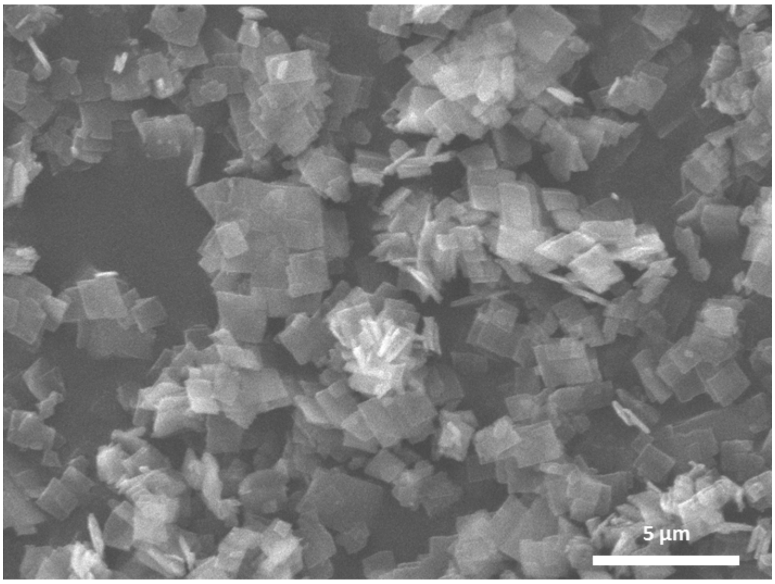

Fig. S4 An SEM of $Cu(OH)_2$-derived $Cu_2(bdc)_2(bpy)$, showing a thin, plate-like morphology.

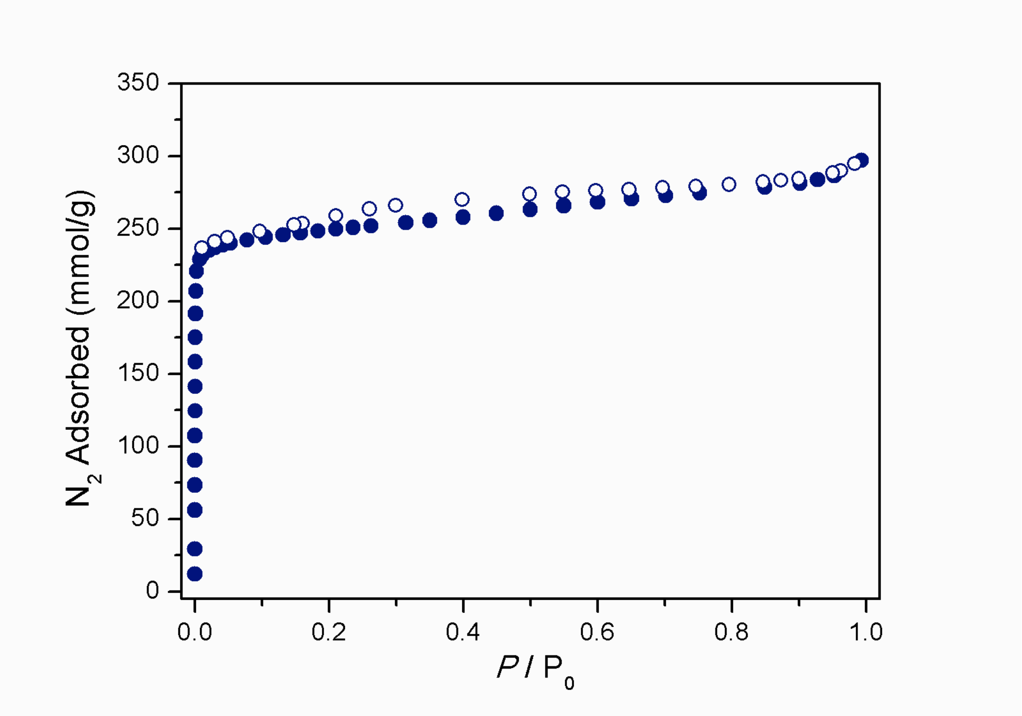

Fig. S5 N_2 adsorption data for $Cu(OH)_2$-derived $Cu_2(bdc)_2(bpy)$ collected at 77 K. Closed and open symbols represent adsorption and desorption, respectively.

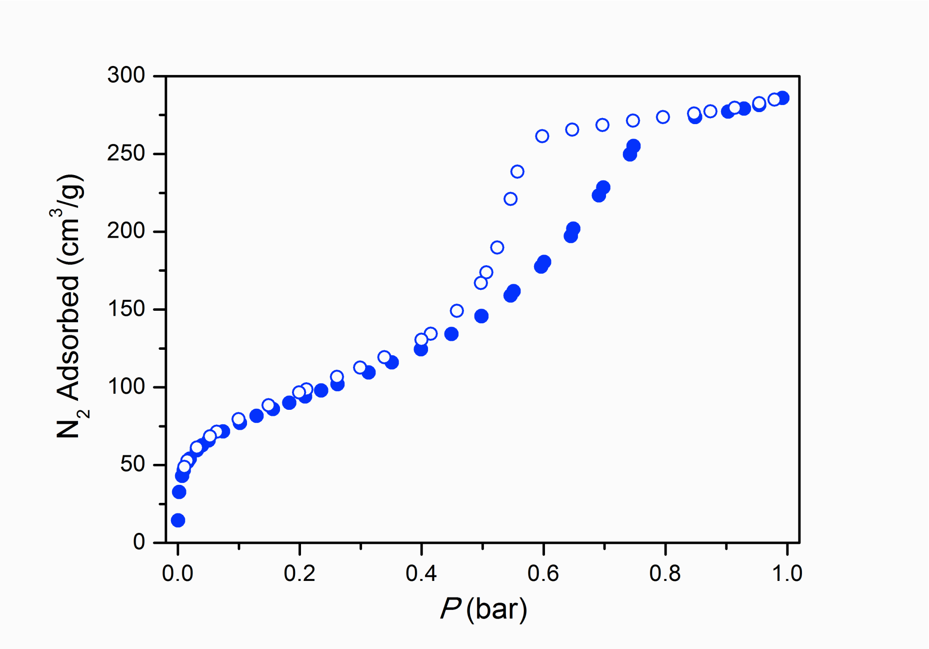

Fig. S6 N_2 adsorption data for the parent $Cu(OH)_2$-PAAm composite collected at 77 K. Closed and open symbols represent adsorption and desorption, respectively.

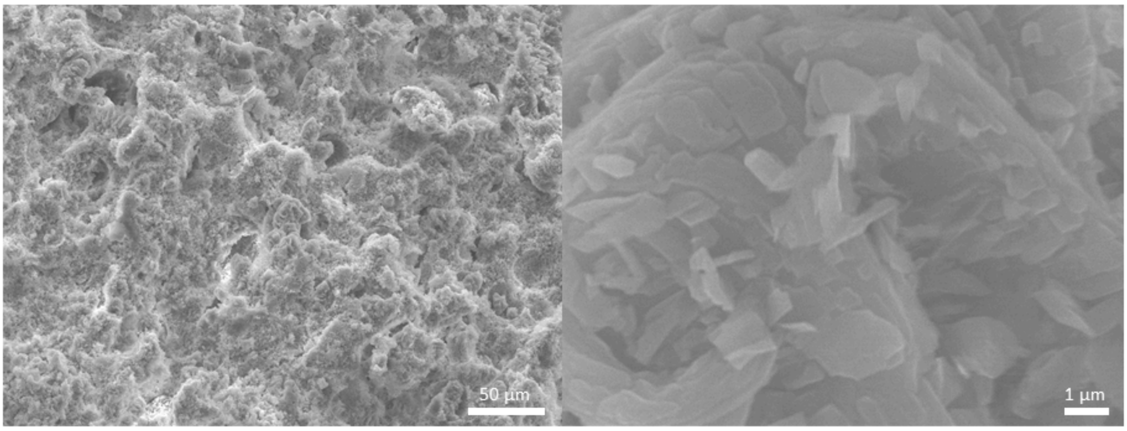

Fig. S7 SEM images of a cross section of a $Cu_2(bdc)_2(MeOH)_2$ monolith after replication showing a (left) a wide view and (right) a zoomed-in view.

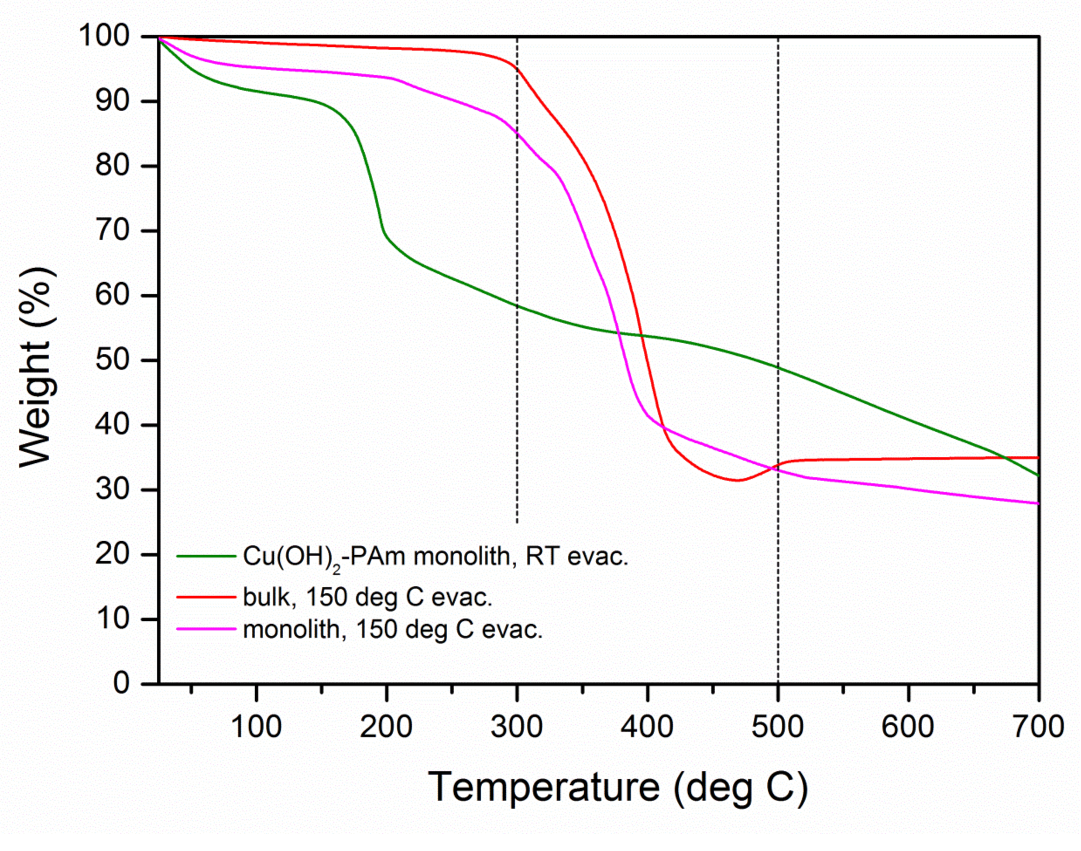

Fig. S8 Thermogravimetric data for the parent $Cu(OH)_2$-PAAm monolith (green), bulk Cu(bdc)(MeOH) (red), and the Cu(bdc)(MeOH)-PAAm monolith (pink) collected under an N_2 flow using a temperature ramp rate of 2 K/min. The dotted line represents the weight transition used to estimate the composition of the replicated monolith.

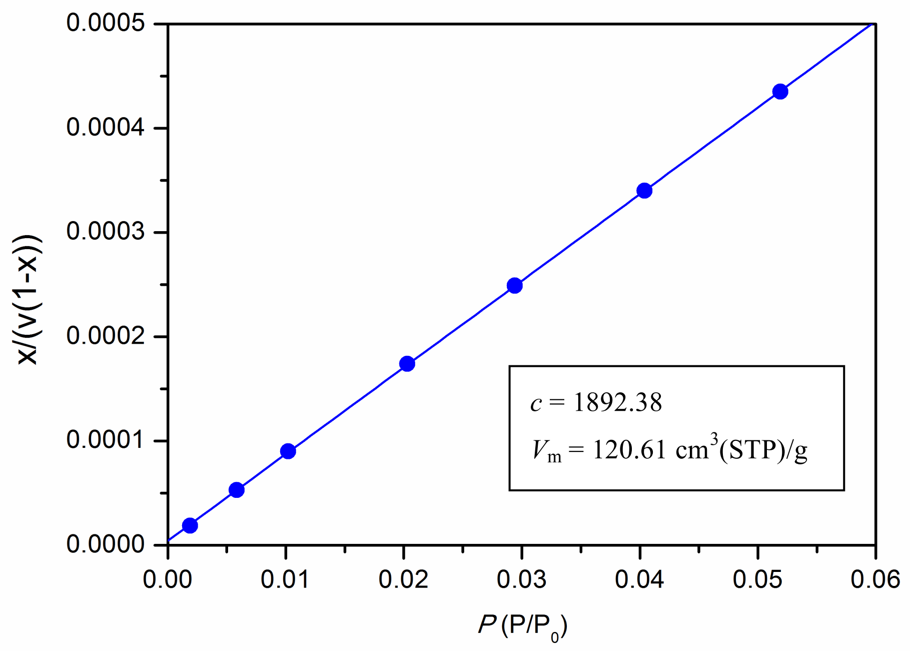

Fig. S9 A BET plot of the adsorption isotherm for N_2 in the Cu(bdc)(MeOH)-PAAm monolith at 77 K, where *x* represents the quantity (P/P_0) and *V* is the volume of N_2 adsorbed. The blue line represents a linear best fit of the data points. Inset: parameters for the linear best fit and resulting constants for calculation of the BET surface area.

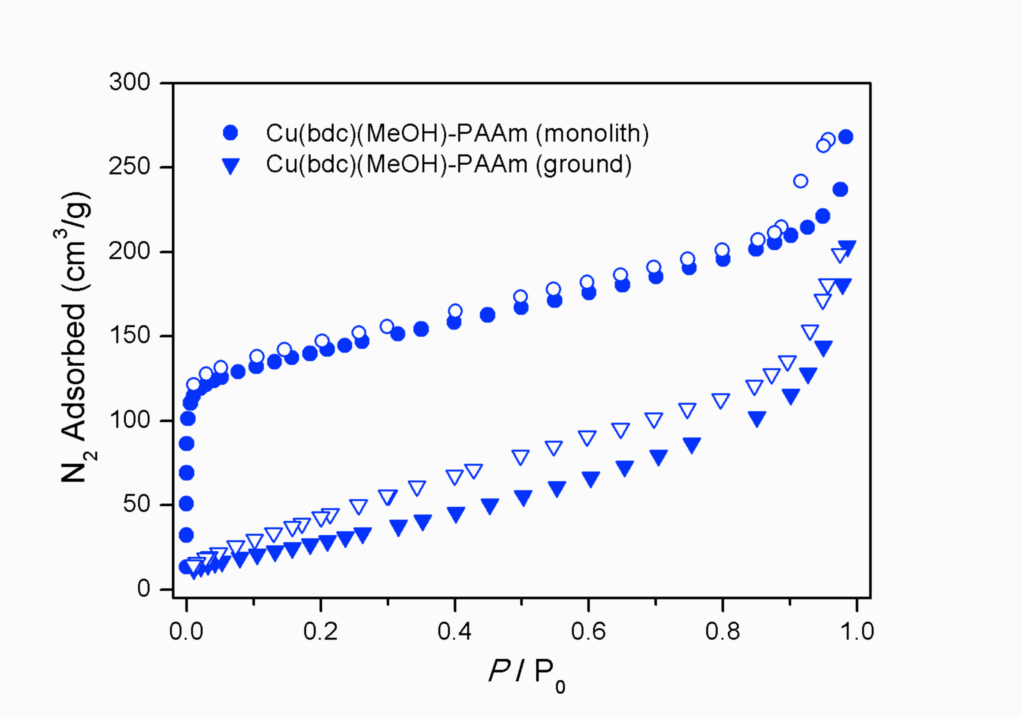

Fig. S10 N_2 adsorption data collected at 77 K for the $Cu_2(bdc)_2(MeOH)_2$ replicate in monolith form (circles) and after mechanical grinding (triangles). Closed and open symbols represent adsorption and desorption, respectively.

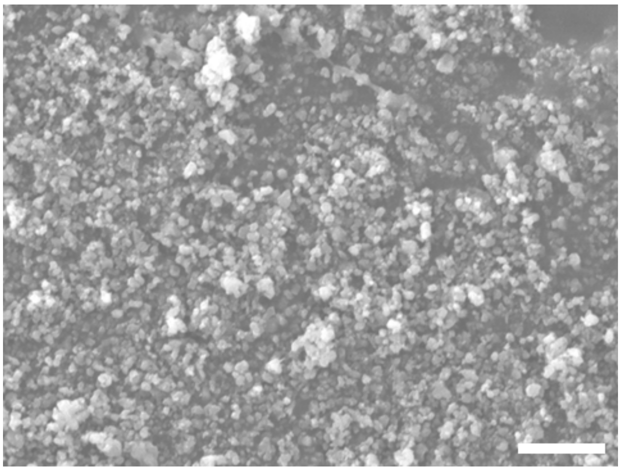

Fig. S11 Field-emission SEM image of the $Cu_2(bdc)_2(MeOH)_2$ monolith after mechanical grinding. The scale bar represents a distance of 10 μm.

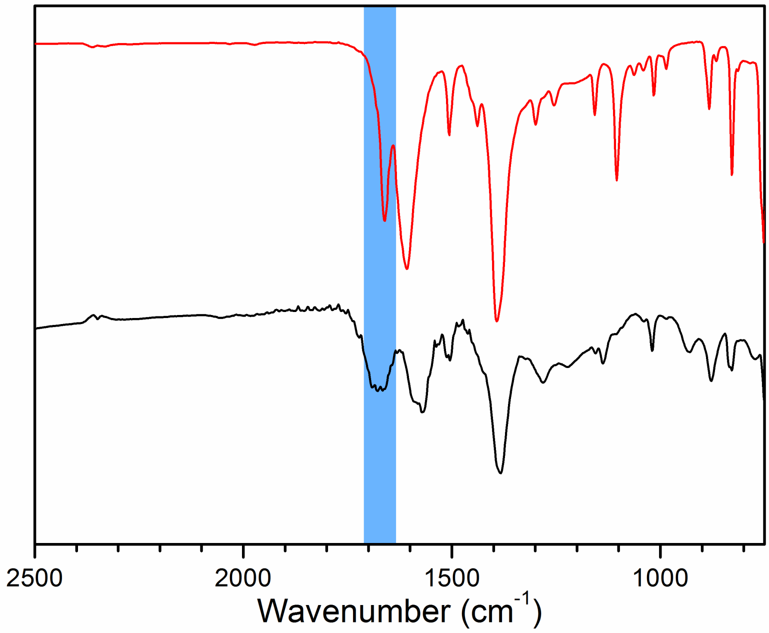

Fig. S12 Infrared spectra for a $Cu_2(bdc)_2(MeOH)_2$ monolith prepared by coordination replication (black), and a $Cu_2(bdc)_2(MeOH)_2$ powder prepared by reaction of a mechanically ground sample of the same parent phase (red). The shaded region indicates the amide C=O stretch, which reflects the presence of polyacrylamide within the sample.

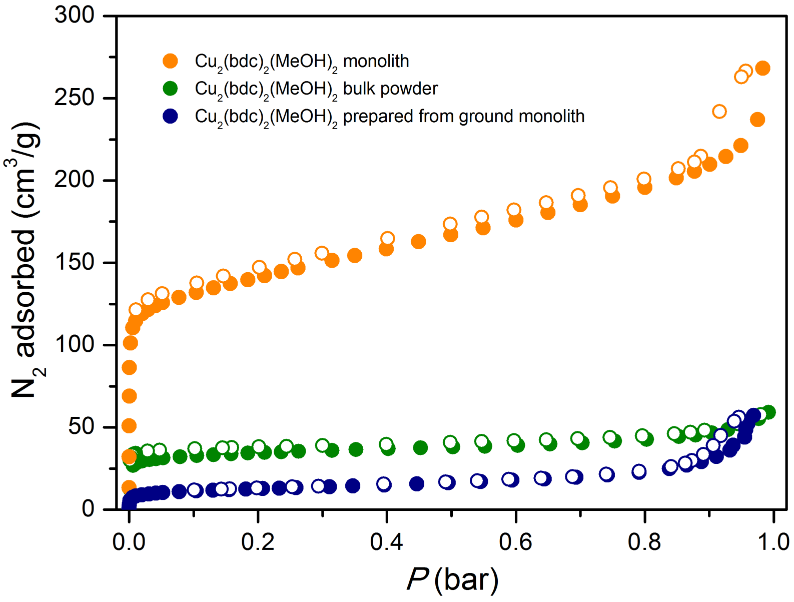

Fig. S13 N_2 adsorption isotherms collected at 77 K for a $Cu_2(bdc)_2(MeOH)_2$ bulk powder (green), a $Cu_2(bdc)_2(MeOH)_2$ monolith (orange), and a $Cu_2(bdc)_2(MeOH)_2$ powder sample prepared from a ground sample of the parent monolith (blue). Closed and open symbols represent adsorption and desorption data, respectively.

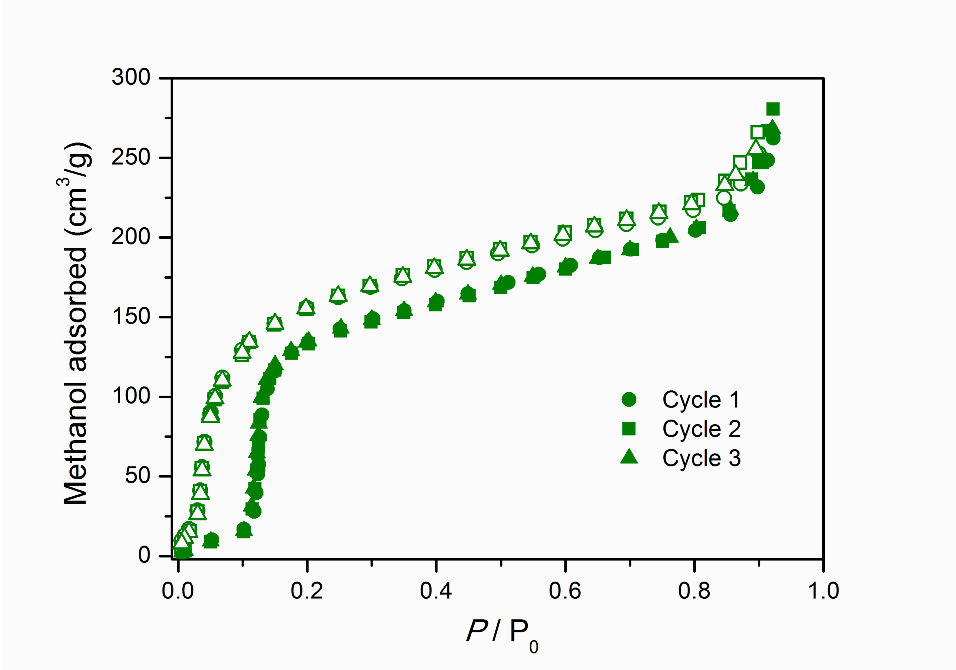

Fig. S14 MeOH adsorption data for a monolithic $Cu_2(bdc)_2(bpy)$ replicate at 298 K for three adsorption and desorption cycles. Closed and open symbols represent adsorption and desorption, respectively.

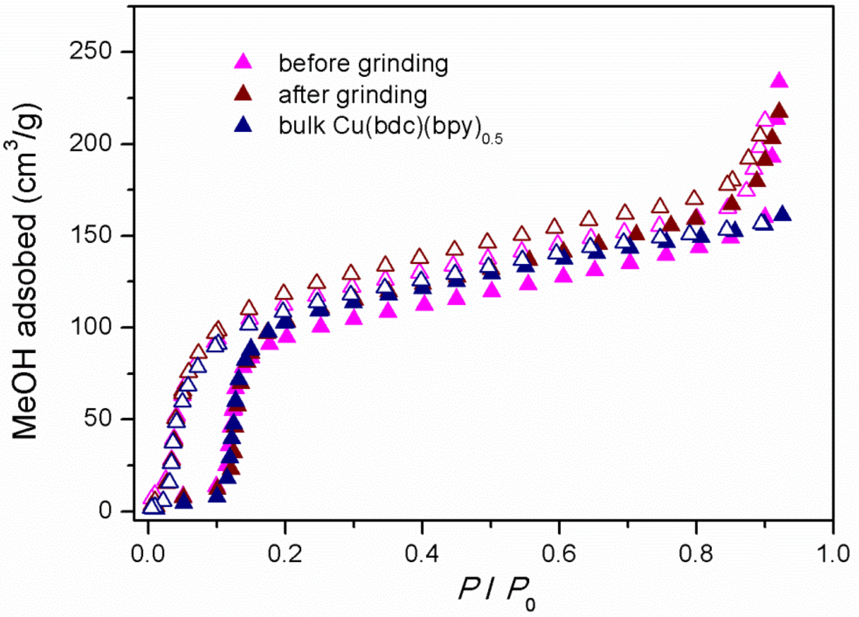

Fig. S15 MeOH adsorption data collected at 298 K for the $Cu_2(bdc)_2(bpy)$ replicate in monolithic form (pink) and after mechanical grinding (brown), and a bulk $Cu_2(bdc)_2(bpy)$ sample prepared from $Cu(OH)_2$ (blue). Closed and open symbols represent adsorption and desorption, respectively.

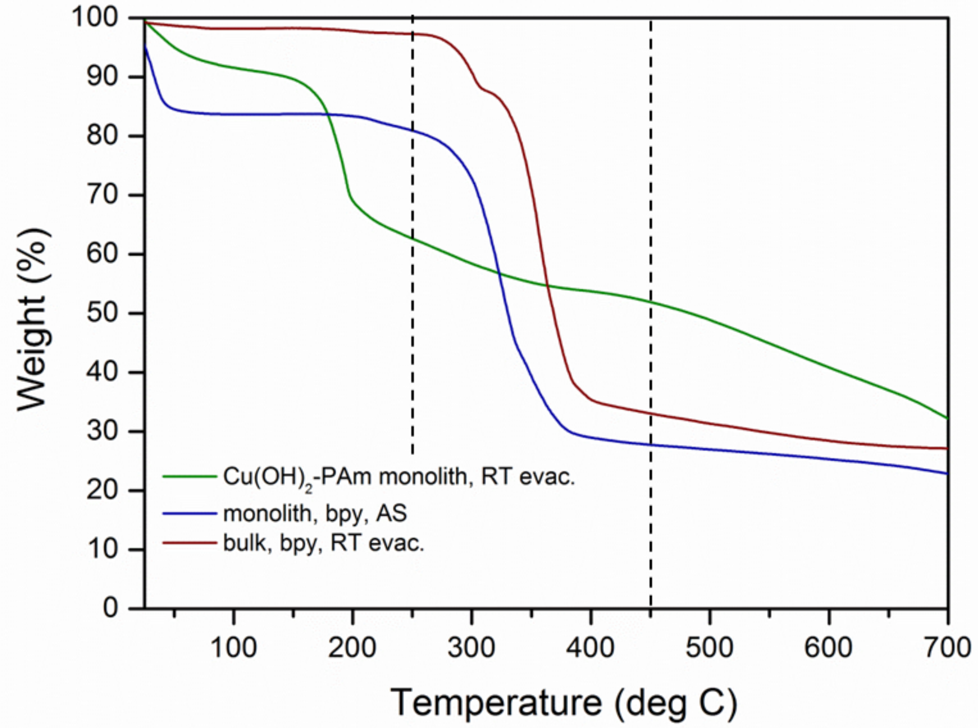

Fig. S16 Thermogravimetric data for the parent $Cu(OH)_2$-PAAm monolith (green), bulk $Cu(bdc)(bpy)_{0.5}$ (brown), and the $Cu(bdc)(bpy)_{0.5}$-PAAm monolith (blue) collected under an N_2 flow using a temperature ramp rate of 2 K/min. The dotted line represents the weight transition used to estimate the composition of the replicated monolith.

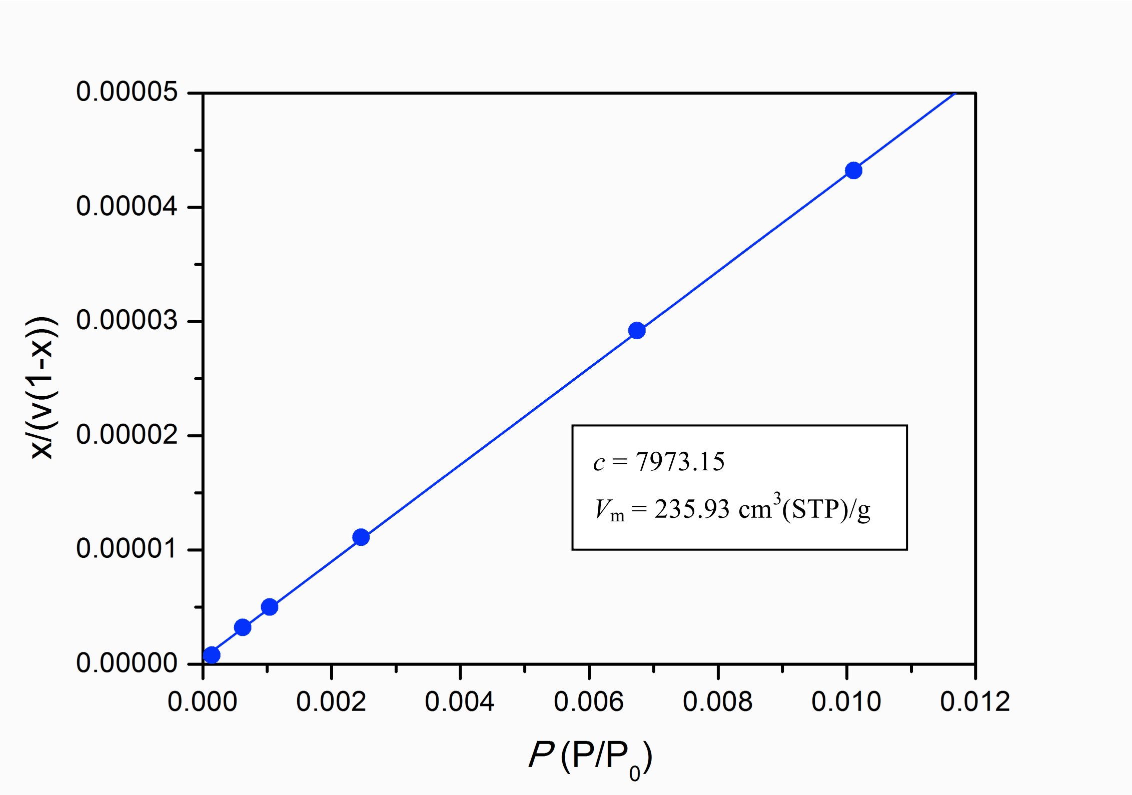

Fig. S17 A BET plot of the adsorption isotherm for N_2 in bulk Cu(bdc)(bpy)$_{0.5}$ prepared from $Cu(OH)_2$ at 77 K, where *x* represents the quantity (P/P_0) and *V* is the volume of N_2 adsorbed. The blue line represents a linear best fit of the data points. Inset: parameters for the linear best fit and resulting constantsfor calculation of the BET surface area.

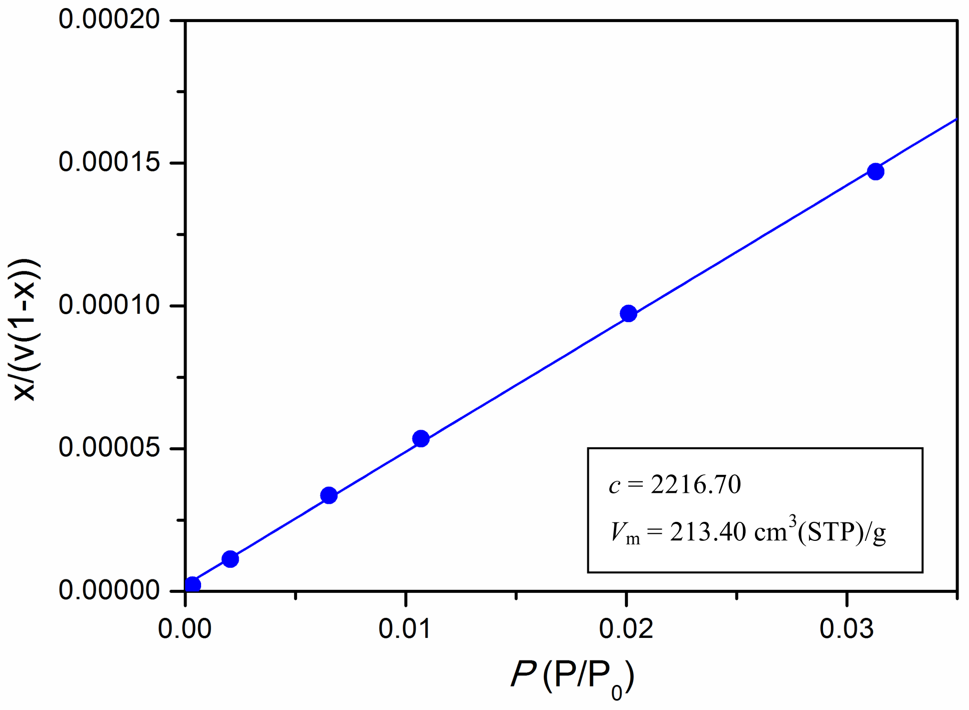

Fig. S18 A BET plot of the adsorption isotherm for N_2 in the Cu(bdc)(bpy)$_{0.5}$-PAAm monolith at 77 K, where *x* represents the quantity (P/P_0) and *V* is the volume of N_2 adsorbed. The blue line represents a linear best fit of the data points. Inset: parameters for the linear best fit and resulting constants for calculation of the BET surface area.

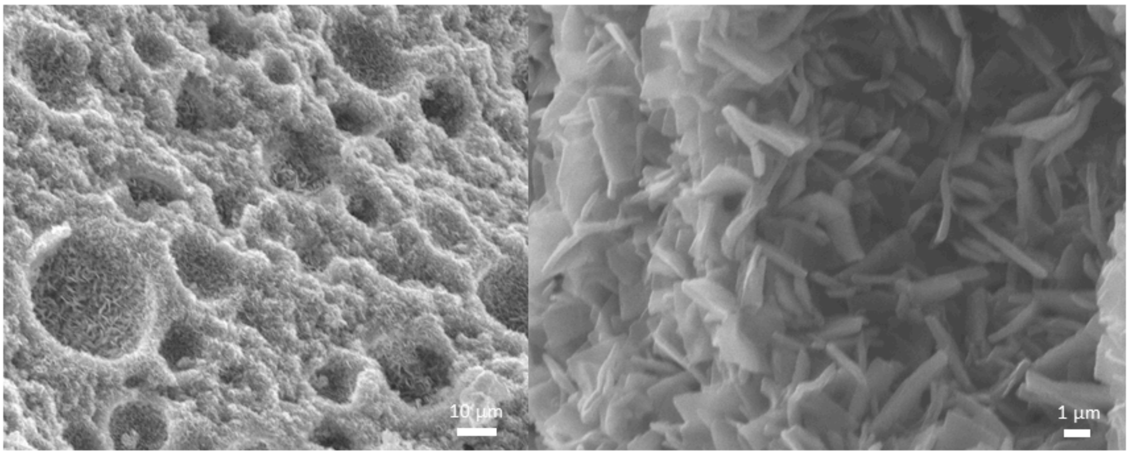

Fig. S19 Field-emission SEM images showing the $Cu_2(bdc)_2(bpy)$ monolith after evacuation-adsorption-evacuation cycling.

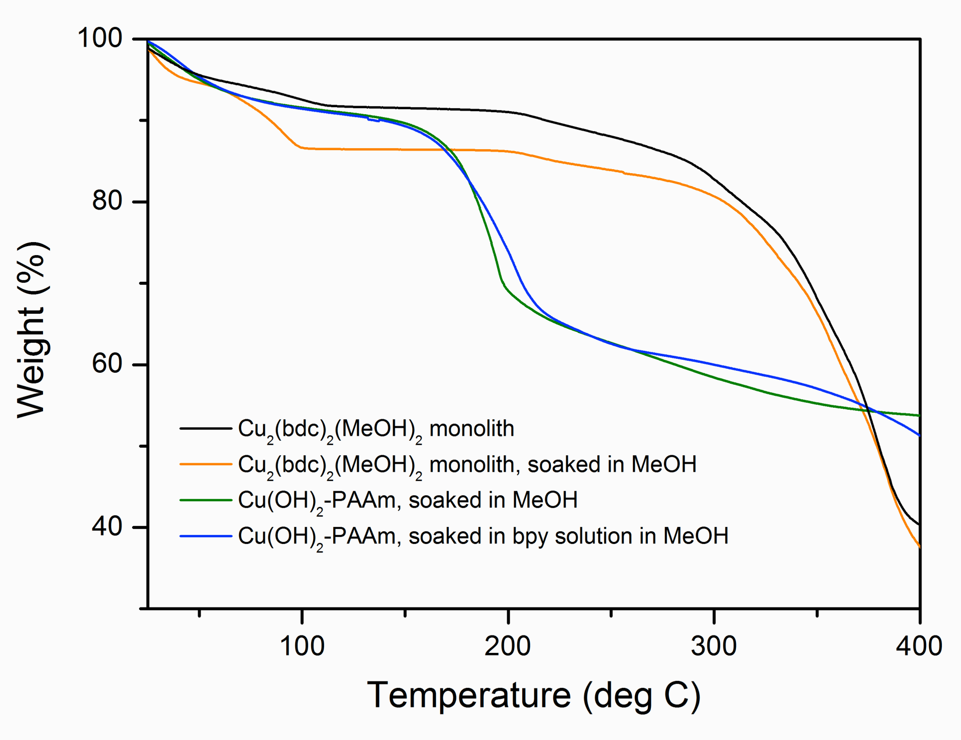

Fig. S20 TGA data for a $Cu_2(bdc)_2(MeOH)_2$ monolith (black) and after soaking in methanol for 2 weeks (orange), and the $Cu(OH)_2$-polyacrylamide monolith soaked in methanol for 2 weeks (green) and in a bpy solution in methanol (blue). Data collected at a ramp rate of 2 K/min.
